# The strength of gut microbiota transfer along social networks and genealogical lineages in the house mouse

**DOI:** 10.1093/femsec/fiae075

**Published:** 2024-05-10

**Authors:** Barbora Bendová, Barbora Vošlajerová Bímová, Dagmar Čížková, Kristina Daniszová, Ľudovít Ďureje, Zuzana Hiadlovská, Miloš Macholán, Jaroslav Piálek, Lucie Schmiedová, Jakub Kreisinger

**Affiliations:** Department of Zoology, Faculty of Science, Charles University, Prague 128 00, Czech Republic; Institute of Vertebrate Biology of the Czech Academy of Sciences, Brno 603 00, Czech Republic; Institute of Animal Physiology and Genetics, Czech Academy of Sciences, Brno 602 00, Czech Republic; Institute of Vertebrate Biology of the Czech Academy of Sciences, Brno 603 00, Czech Republic; Institute of Animal Physiology and Genetics, Czech Academy of Sciences, Brno 602 00, Czech Republic; Institute of Vertebrate Biology of the Czech Academy of Sciences, Brno 603 00, Czech Republic; Institute of Animal Physiology and Genetics, Czech Academy of Sciences, Brno 602 00, Czech Republic; Institute of Animal Physiology and Genetics, Czech Academy of Sciences, Brno 602 00, Czech Republic; Institute of Vertebrate Biology of the Czech Academy of Sciences, Brno 603 00, Czech Republic; Department of Zoology, Faculty of Science, Charles University, Prague 128 00, Czech Republic; Institute of Vertebrate Biology of the Czech Academy of Sciences, Brno 603 00, Czech Republic; Department of Zoology, Faculty of Science, Charles University, Prague 128 00, Czech Republic

**Keywords:** gastrointestinal tract, inter-individual transmission, microbiome, relatedness, social contact

## Abstract

The gut microbiota of vertebrates is acquired from the environment and other individuals, including parents and unrelated conspecifics. In the laboratory mouse, a key animal model, inter-individual interactions are severely limited and its gut microbiota is abnormal. Surprisingly, our understanding of how inter-individual transmission impacts house mouse gut microbiota is solely derived from laboratory experiments. We investigated the effects of inter-individual transmission on gut microbiota in two subspecies of house mice (*Mus musculus musculus* and *M. m. domesticus*) raised in a semi-natural environment without social or mating restrictions. We assessed the correlation between microbiota composition (16S rRNA profiles), social contact intensity (microtransponder-based social networks), and mouse relatedness (microsatellite-based pedigrees). Inter-individual transmission had a greater impact on the lower gut (colon and cecum) than on the small intestine (ileum). In the lower gut, relatedness and social contact independently influenced microbiota similarity. Despite female-biased parental care, both parents exerted a similar influence on their offspring’s microbiota, diminishing with the offspring’s age in adulthood. Inter-individual transmission was more pronounced in *M. m. domesticus*, a subspecies, with a social and reproductive network divided into more closed modules. This suggests that the transmission magnitude depends on the social and genetic structure of the studied population.

## Teaser

The influence of social contacts and relatedness on microbiota is greater in cecum and colon than in ileum; fathers and mothers have a comparable influence on microbiota of the offspring.

## Introduction

The gut microbiota of animals represents a diverse consortium of microorganisms dominated by Eubacteria and their viruses (Vemuri et al. 2020). The extensive functional potential encoded by microbial metagenomes expands the metabolic capabilities of the host and influences various aspects of its phenotype (Heijtz et al. 2011, den Besten et al. 2013, Beura et al. 2016). Unsurprisingly, disruptions in the functions of the gut microbiota, referred to as dysbiosis, often lead to negative health consequences. In modern human societies, the prevalence of various inflammatory and metabolic disorders associated with dysbiosis of the microbiota has significantly increased in recent decades (Cekanaviciute et al. 2017, Manfredo Vieira et al. 2018). Therefore, a crucial objective of current research on host-associated gut microbiota is to comprehend the mechanisms that shape the microbiota during host ontogeny and give rise to variation in its composition.

While a subset of bacteria invades the gut from the environment, a substantial proportion of the gut microbiota consists of bacterial species with highly specific ecological requirements, making them rarely found in environmental sources outside the host body (Li et al. 2016). Direct transfer of these bacteria between host individuals is believed to facilitate their spread within host populations (Tung et al. 2015, Moeller et al. 2016b, Amato et al. 2017). In invertebrates, the connections between bacterial adaptation to obligatory symbiosis and their vertical transmission, often spanning host speciation events, are well known (Moran et al. 2008). In vertebrates, the tightness of the host–microbiota relationship is still largely unexplored. A few empirical studies have only recently uncovered bacterial lineages that have co-diversified with their vertebrate hosts, as well as changes in the phenotype and genome of these bacteria that facilitate their persistence in the host gut and their transmission between host individuals (Moeller et al. 2016a, Waskito and Yamaoka 2019, Karcher et al. 2020, Suzuki et al. 2022).

Vertebrate embryos generally develop in a sterile or near-sterile environment, and the colonization of their bodies by symbiotic bacteria begins as early as hatching or birth (Perez-Muñoz et al. 2017, Kennedy et al. 2023, Těšický et al. 2024). Parental transmission of bacteria is an important pathway that facilitates the establishment of a normal microbiota in newborns. Inoculation by maternal vaginal microbiota during delivery is likely the most significant initial source of gut bacteria in therian (viviparous) mammals (Dominguez-Bello et al. 2010, Ferretti et al. 2018). However, other forms of physical contact with parents are also likely to facilitate the establishment of microbial populations in vertebrate juveniles. This is supported by the similarity in microbiota between parents and offspring of oviparous vertebrates (Kreisinger et al. 2017, Kouete et al. 2023), where direct transfer of maternal microbiota during birth or breastfeeding is not possible. In addition to vertical transmission of gut microbiota along host genealogical lineages mediated by contacts with parents, social contacts with distantly related conspecifics represent another channel of gut microbiota transmission. This is supported by many studies, in which the intensity of social interactions between individuals predicts the similarity of their gut microbiota (Lax et al. 2014, Tung et al. 2015, Amato et al. 2017, Dill-McFarland et al. 2019, Sarkar et al. 2020, Raulo et al. 2021).

The bacteria transmitted and incorporated into the vertebrate gut immediately affect the community structure, but they can also exert indirect effects that manifest with a time delay. In this regard, considerable attention has been paid to the role of acquired bacteria in the subsequent succession and maturation of the gut community in early life humans (reviewed in Wang et al. 2020). However, studies directly quantifying the changes in the strength of transmission effects over time are rare. For example, in humans, the effect of cesarean section, which prevents the transfer of vaginal microbiota to offspring, is strongest in neonates up to a few months old, but significant differences due to delivery mode persist until several years of age (Roswall et al. 2021). In birds, social transmission of microbiota during copulations leads to a correlated composition of cloacal microbiota between sexual partners (White et al. 2010, Kreisinger et al. 2015). Nevertheless, the similarity decreases rapidly after a few days when copulation is experimentally restricted (White et al. 2010), suggesting that the effect of transmission is only transient.

A limitation of our current knowledge is that all studies on gut microbiota transmission to date have focussed exclusively on the communities of the lower intestinal segments (i.e. colon and cecum), while the impact of inter-individual transmission on microbiota in other gut sections remains completely unknown. There is compelling evidence that the gut microbiota outside the lower intestine differs from the lower gut microbiota in its composition and its effects on host physiology and immune functions (Zoetendal et al. 2012, Kastl et al. 2020). While ecological gradients along the gut appear to play a major role in these differences between gut communities (Stearns et al. 2011), the mode of bacterial transmission can represent yet another important factor affecting the assembly and turnover of the local microbiota, similar to what has been shown for gut vs. oral communities (Valles-Colomer et al. 2023).

Furthermore, there is a lack of suitable model systems to study the effects of microbiota transmission. While studies in wild vertebrate populations are crucial, collecting data on the inter-individual variability of the microbiota that allows discrimination of sources of microbiota transmission (i.e. parents, conspecifics, and environment) can be extremely challenging. Another caveat is that microbiota transmission itself is not the only factor affecting microbiota variation between individuals. Factors such as diet composition, genetically encoded regulatory mechanisms of the host, and the host’s health and ontogenetic state can either restrict or promote the growth of transmitted bacteria in the gut (Hasan and Yang 2019). Typically, these factors show high variation in wild populations and are difficult to determine.

At the same time, captive-bred model animals may not be optimal for this type of research. A prime example is the laboratory house mouse, which is an important model in various areas of biomedical research, including the effects of gut microbiota on the host (Ericsson and Franklin 2021). However, the specific pathogen-free mice used for this purpose have a highly altered microbiota compared to their wild ancestors, with dramatic effects on some of their phenotypic traits (Rosshart et al. 2017), raising concerns about the general relevance of this model. The recent call for a mouse model with more realistic host–symbiont interactions has led to the concept of “re-wildering” laboratory mice (Graham 2021). For the successful application of such a concept, it is necessary to understand the mechanisms that lead to the assembly of microbial communities in the house mouse under natural conditions.

We investigated the transmission of gut bacteria between individuals of two closely related house mouse subspecies (*Mus musculus musculus* and *M. m. domesticus*, hereafter referred to as MMM and MMD, respectively). We compared the transmission patterns among three gut sections: the colon, the cecum, and the ileum. The mice used in this study were first generation offspring from free-living populations, possessing wild-type microbiota, and were maintained for several generations in semi-natural arena experiments that mimicked free-living populations (Bendová et al. 2022, Mikula et al. 2022). Unlike experiments conducted in laboratory settings, the mating of mice and their social contacts were not constrained by external factors. The common garden setup ensured a homogeneous environment, including a uniform diet. This reduced the chances that microbiota variability was causally driven by factors that varied spatially and directly influenced microbiota composition. The absence of emigration and immigration from and into the studied population allowed detailed analyses of genetic parentage (based on microsatellite data) and social interactions (using radiofrequency identification microtransponders). In this way, the transmission of the microbiota along social networks and genealogical host lineages (Mikula et al. 2022) could be tracked with a degree of precision and completeness that would be nearly impossible to achieve in free-living populations of this species.

Notably, the two host subspecies in the experiment differed in their social and reproductive patterns. Specifically, MMD displayed more “modular” social networks represented by closer and more separated units compared to MMM. Since the “modules” were identified with reproductive units (demes), it was concluded that the experimental MMD populations had more “demic” social structure than MMM. This finding was also consistent with a higher frequency of multiple paternity in the latter subspecies (Mikula et al. 2022). This provided a unique opportunity to assess the impact of this variation on the patterns of inter-individual transmission of gut microbiota.

Our main objectives were as follows: (i) to compare the strength of inter-individual transmission of gut bacteria between closely related and less related individuals in different gut segments and in two house mouse subspecies, (ii) to identify specific bacteria whose abundance variation is explained by social contacts and host genetic relatedness, and (iii) to analyze the temporal persistence of maternal and paternal microbiota transmission.

## Methods

### Data collection

We collected data from 198 house mouse individuals sampled as part of the study conducted by Mikula et al. (2022), which aimed to compare the social and reproductive structure between two house mouse subspecies bred under semi-natural conditions in a common garden setup. Detailed information about the 2014 experiment, which is analyzed in this work, can be found in Mikula et al. (2022). A subset of the adult individuals from this experiment was previously analyzed by Bendová et al. (2022) to assess microbiota divergence between the two mouse subspecies.

Briefly, we used first-generation mice born in captivity, representing the G1 progeny of wild mice from two populations of each subspecies. At the beginning of the ∼9-month-long experiment, 12 MMM and 12 MMD individuals were released in two adjacent enclosures (4 × 2 m each) equipped with six nest boxes. After ∼6 months, the two enclosures were interconnected with a short tunnel [see Fig. S1 in Mikula et al. (2022)]. The mice were kept under constant conditions, including a light–dark regime of 14:10 h and a temperature of ∼20°C. They had access to standard food pellets and water *ad libitum*. The enclosures were regularly checked every 3 days, and newborns were marked by toe-clipping, with their birth dates recorded. Each mouse was injected with a radiofrequency identification microtransponder to monitor their movements. The entrance of each nest box and the tunnel connecting the enclosures were each equipped with two transponder readers. At the end of the experiment, all mice were sacrificed by cervical dislocation and immediately dissected. Tissue samples were collected from three gut sections: 1 cm of the distal part of the ileum, the entire cecum, and the colon. Each sample was placed in a sterile cryotube, snap-frozen in liquid nitrogen, and stored at −80°C until analyzed. During the dissections, only flame-sterilized tools were used, and special care was taken to avoid potential sample contamination.

### Intensity of social interactions and level of genetic relatedness

Mouse DNA was extracted from ethanol-preserved toe clips using the Qiagen DNeasy 96 Blood & Tissue Kit. A panel of 25 microsatellites (Mikula et al. 2022) was used for parentage assignment. The parentage analysis was performed using CERVUS v. 3.0.3 (Kalinowski et al. 2007) at a 95% confidence level. Individuals who met the defined requirements, such as reaching sexual maturity at the estimated time of conception, were included in the analysis as candidate parents. The parentage data were analyzed using the R package *AGHmatrix* (Amadeu et al. 2016) to reconstruct MMM and MMD genealogies, and relatedness coefficients between individuals were estimated. Importantly, we did not detect any hybrid between MMM and MMD.

Matrices describing the intensity of social contacts between MMM and MMD individuals were constructed as described in Mikula et al. (2022). In brief, the social contact between two individuals was measured as the time they spent together in any of the nest boxes, regardless of other individuals potentially present in the nest boxes. A visit to a nest box was defined as the time between entering (recorded as a twofold signal: the first from the outer reader, followed by that from the inner reader) and leaving the nest (recorded in reverse order, i.e. inner—outer reader). Consequently, in our study, we define social interactions based on the spatio-temporal co-occurance of two individuals. During such encounters, multiple behavioral interactions and associated types of physical contact could take place. However, our experimental design did not allow us to observe and distinguish between them.

### 16S rRNA sequencing of gut microbiota

Metagenomic DNA from gut tissue samples was extracted using the PowerSoil DNA Isolation Kit (QIAGEN). Sequencing libraries were prepared using a two-step Polymerase Chain Reaction (PCR) protocol as described in Čížková et al. (2021). In the first PCR, standard metabarcoding primers of Klindworth et al. (2013) targeting the V3–V4 region of the bacterial 16S rRNA gene (S-D-Bact-0341-b-S-17: CCTACGGGNGGCWGCAG and S-D-Bact-0785-a-A-21: GACTACHVGGGTATCTAATCC) were used. These primers were extended by inline barcodes and priming sites for the second PCR. In the second PCR, dual indexes were introduced, and Illumina-compatible Nextera-like sequencing adapters were added. Each metabarcoding PCR was performed in technical duplicate to account for PCR and sequencing stochasticity. The PCR products were pooled based on their concentration and sequenced using an Illumina MiSeq platform (v3 kit, 300 bp paired-end reads) at CEITEC, Brno, Czech Republic.

The resulting fastq files were demultiplexed, and 16S rRNA primers were detected and trimmed using *Skewer* (Jiang et al. 2014). R package *dada2* (Callahan et al. 2016) was used to eliminate low-quality reads (expected error rate per paired-end read >1), to denoise the quality-filtered reads, and to generate abundance matrices of read counts for each 16S rRNA sequencing variant (hereafter ASV) in each sample. Denoising was performed with the pool=”pseudo” option to increase the sensitivity of detection of rare ASVs in samples with low sequencing coverage (Bardenhorst et al. 2022). Subsequently, *uchime2* (Edgar 2016 et al. 2016) was used to detect and eliminate chimeric ASVs with Silva database v.138 (Quast et al. 2013) as a reference for filtering the chimeras. Taxonomy assignment for non-chimeric ASVs was performed with 80% posterior confidence using the RDP classifier (Wang et al. 2007). The Silva database v.138 (Quast et al. 2013) was used for bacterial ASV annotations. The consistency of ASV profiles between technical duplicates was checked using Procrustes analysis (Legendre and Legendre 1998). The duplicated data were then merged, eliminating any ASVs that were not detected in both duplicates.

### Statistical analyses

We employed linear mixed-effects models (LMMs) to assess the strength of the association between microbiota composition, genetic relatedness, and intensity of social contacts. To characterize the variation in microbiota composition among samples, we calculated Bray–Curtis dissimilarities (based on the relative abundance of ASVs) and Jaccard dissimilarities (based on the presence/absence of ASVs). Phylogenetically weighted distances (e.g. UniFrac), which downweight compositional divergence caused by closely related bacterial ASVs, were not considered. Such a feature is not desirable for our study, as phylogenetically related bacteria may have similar properties (e.g. oxygen tolerance or spoluration ability) that facilitate their dispersal (Galperin et al. 2012).

Bray–Curtis and Jaccard dissimilarities were then transformed into similarity indices using the formula (f1): *S*[*i, j*] = 1–*D*[*i, j*], where *S* and *D* represent the similarity and dissimilarity between the *i*-th and *j*-th samples, respectively. The logit-transformed values of the similarity indices were used as response variables in LMMs, assuming a Gaussian error distribution. To account for uneven sequencing depth, the ASV abundance matrix was rarefied before calculating microbiota dissimilarities. The rarefaction thresholds were set to the minimum sequencing depth reached (i.e. 2374 reads). To address the non-independence of pairwise (dis)similarity values, we employed the R package *lmerMultiMember* (van Paridon et al. 2022), which allowed us to include pairs of mouse individuals associated with each similarity value as a multigroup membership random effect. The models were fitted separately for each gut section. As predictors in the LMMs, we considered the effect of host subspecies identity, the intensity of social contacts between host individuals, their relatedness coefficients, and interactions between the latter two variables and subspecies identity. Stepwise backward elimination of nonsignificant terms from the full model was performed with *step.lmerModLmerTest* function from the package *lmerTest* (Kuznetsova et al. 2017), and likelihood ratio tests were used to determine the significance of the predictors. The proportion of variance explained by the effects of relatedness or social contacts was calculated using the R package *variancePartition* (Hoffman and Schadt 2016).

ASVs whose between-sample variation was predicted by the effect of relatedness or social interactions were identified using Procrustes analysis. The read counts in the ASV abundance matrix were transformed to a centered log ratio (clr) (Aitchison 1982) using R package compositions (van den Boogaart and Tolosana-Delgado 2008). Pairwise absolute differences in clr values between all samples were calculated for each ASV included in the analyses. The resulting dissimilarity matrices were scaled using principal coordinate analysis (PCoA) with Cailliez correction for negative eigenvalues using R package ape (Paradis and Schliep 2019) and resulting PCoA scores were used as responses in Procrustes analyses (conducted using function from R pacakge vegan; Oksanen et al. 2020). Matrices containing relatedness coefficients and social interaction intensity were inverted using the f1 formula, scaled using PCoA, and the resulting PCoA scores were used as response variables in Procrustes analyses. ASV-level analyses were conducted separately for each host subspecies and gut section, using a subset of ASVs present in at least 20 samples of a gut section. The false discovery rate (FDR) method by Benjamini and Hochberg (1995) was employed to account for false-positive detections due to multiple testing, and results with FDR values <0.05 were considered significant.

We hypothesized that the strength of parental effects on the offspring microbiota is not constant over time, but gradually decreases after the offspring achieve independence from parental care. To test whether the strength of parental effects on offspring microbiota varies with offspring age, we calculated microbiota similarities between parents and their genetic offspring for all gut sections, and included them as a response in LMMs. Offspring age, sex, gut sections, subspecies, and their interactions were used as predictors. LMMs included parental and offspring identity as multigroup membership random effects. The nonlinear effect of offspring age on dissimilarity with parents was modeled using natural cubic splines. To determine the optimal complexity of the model fit, linear models (estimating the intercept and slope for the effect of offspring age) were compared with models including nonlinear splines with degrees of freedom ranging from two to four, using the Akaike information criterion. Temporal changes in similarity in microbiota composition between offspring from the same litter were analyzed in the same manner as described above, but the effect of sex was not considered because it varied between sibling pairs. To compare the patterns of microbiota dissimilarity between parents and offspring, and between offspring from the same litter, with the overall dissimilarity of microbiota composition, we estimated the average population-wide dissimilarity between genetically unrelated individuals (relatedness coefficients < 0.25) for each intestinal section using LMMs with multigroup membership random effects. In all LMMs, microbiota dissimilarities were logit-transformed (Warton and Hui 2011) to achieve a normal distribution of residuals.

It is essentially unclear how similarity in microbiota composition, due to genetic relatedness or the intensity of social interactions, translates into alpha diversity at the individual level. Since we are unable to formulate plausible hypotheses for microbial alpha diversity, we intentionally did not conduct thorough analyses of alpha diversity in our study.

## Results

### Gut microbiota variation

We analyzed a total of 585 microbiota samples (Table S1, Supplementary Data) from three gut sections (ileum, cecum, and colon) of 198 house mice belonging to two subspecies (52 males and 45 females of MMM, and 51 males and 50 females of MMD). Two colon, two cecum, and five ileum samples were not included into analyses due to missing sample or possible contamination during the DNA isolation. The sequencing data included 8 065 266 high-quality reads, with an average sequencing depth per sample of 13 787 (range 2374–45 661). We detected a total of 3136 non-chimeric bacterial ASVs.

Ileum exhibited the lowest alpha diversity and displayed a distinct microbiota composition compared to the cecum and colon. Furthermore, the ileum differed in terms of its taxonomic content. PCoA revealed clear differences in microbiota composition between the two house mouse subspecies (Fig. S1, Supplementary Data).

### Effect of social contacts and relatedness on microbiota similarity

**Figure 1.**
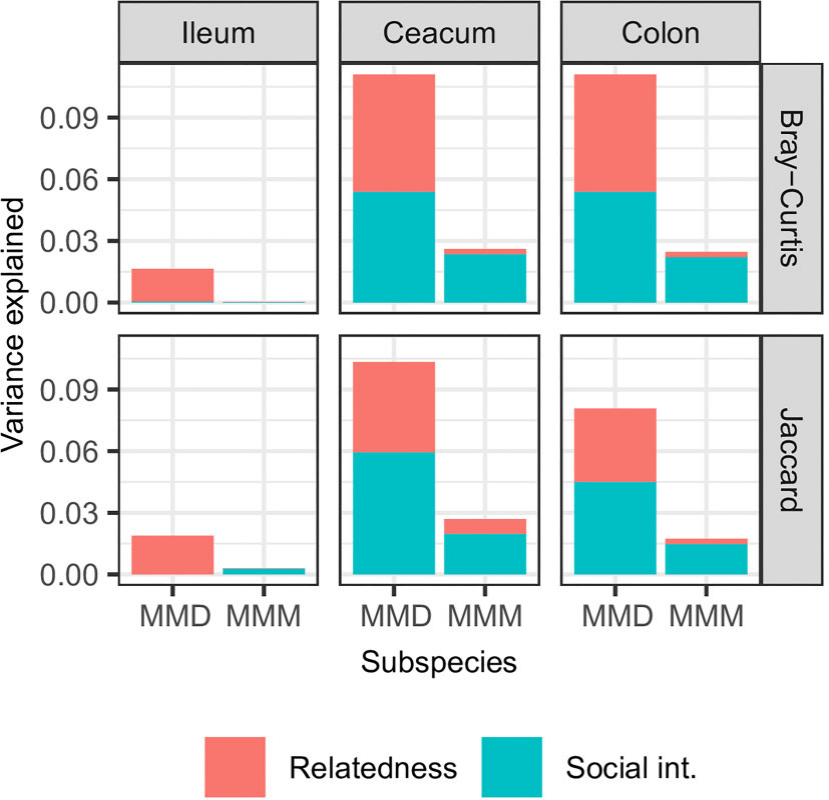
Proportion of variation in microbiota composition explained by social contacts and relatedness. Estimates were performed separately for each gut section (ileum, cecum, colon), each mouse subspecies (MMD, MMM), and for two microbiota similarity indices (Bray–Curtis, Jaccard) using linear mixed-effects models.

The effects of social contacts and relatedness varied significantly in their relative importance between the two host subspecies, as indicated by their significant interactions with subspecies identity (Table S2, Supplementary Data). Subsequent LMMs conducted on data subsets corresponding to the three gut sections in each mouse subspecies revealed significant influences of both social contacts and relatedness on the similarity of gut microbiota in the cecum and colon (Table S3, Supplementary Data). These two factors explained a greater proportion of the variation in gut microbiota in MMD compared to MMM. The combined effect of relatedness and social contacts accounted for 8%–10% of the total variation in MMD, whereas it was <3% in MMM (Fig. 1). In the case of the ileum microbiota, the effect of relatedness was only significant for MMD (for both Bray–Curtis and Jaccard similarities) and the effect of social interaction was only significant for MMM and the model with Jaccard but not Bray–Curtis similarities (Table S3, Supplementary Data).

To explore the possibility that the effects of inter-individual transmission of the microbiota might be restricted to close family members that are genetically highly related, we conducted additional analyses by excluding dissimilarities related to full siblings and mother–offspring or father–offspring pairs. Subsequently, we re-ran the aforementioned analyses. The results showed that the microbiota variation explained by both social contacts and relatedness decreased, but remained significant for all gut sections of MMD. In contrast, no significant effect of relatedness was found in MMM (Fig. S2 and Table S3, Supplementary Data). This finding shows that in MMD, the transmission of microbiota occurs along genealogical lineages, while in MMM the social transmission dominates in less related individuals.

The analyses at the level of individual bacteria included 446, 471, and 81 ASVs for the cecum, colon, and ileum, respectively, that were detected in at least 20 individuals. A significant effect of genetic relatedness was found in 29.1% of ASVs from the cecum, 24.0% of ASVs from the colon, and 11.1% of ASVs from the ileum. Social contacts had an effect in 7.2% of ASVs from the cecum, 1.2% from the colon, and 0% from the ileum.

In both house mouse subspecies, social contacts exhibited a congruent effect on ASVs variation. Specifically, we observed a significant correlation between the Procrustes sums of squares (inversely related to the strength of the correlation between the intensity of social contacts and the similarity of abundances of individual ASVs) calculated separately for the two mouse subspecies (Spearman correlation: rho = 0.2972, *P* < 0.0001 for colon, rho = 0.3215, *P* < 0.0001 for cecum, and rho = 0.2650, *P* = 0.0171 for ileum). These findings suggest that individual gut bacteria consistently exhibited dependence or independence on social contacts regardless of the host’s genetic background. In the colon and cecum, we also found a significant correlation in the Procrustes sums of squares between MMM and MMD for the effect of genetic relatedness (Spearman correlation: rho = 0.1986, *P* < 0.0001 for the colon and rho = 0.2416, *P* < 0.0001 for the cecum), although the strength of these correlations was lower compared to social contacts. However, there was no significant agreement between the two mouse subspecies in the case of ileum ASVs correlated to host genetic relatedness (Spearman correlation: rho = −0.0496, *P* = 0.6593).

**Figure 2.**
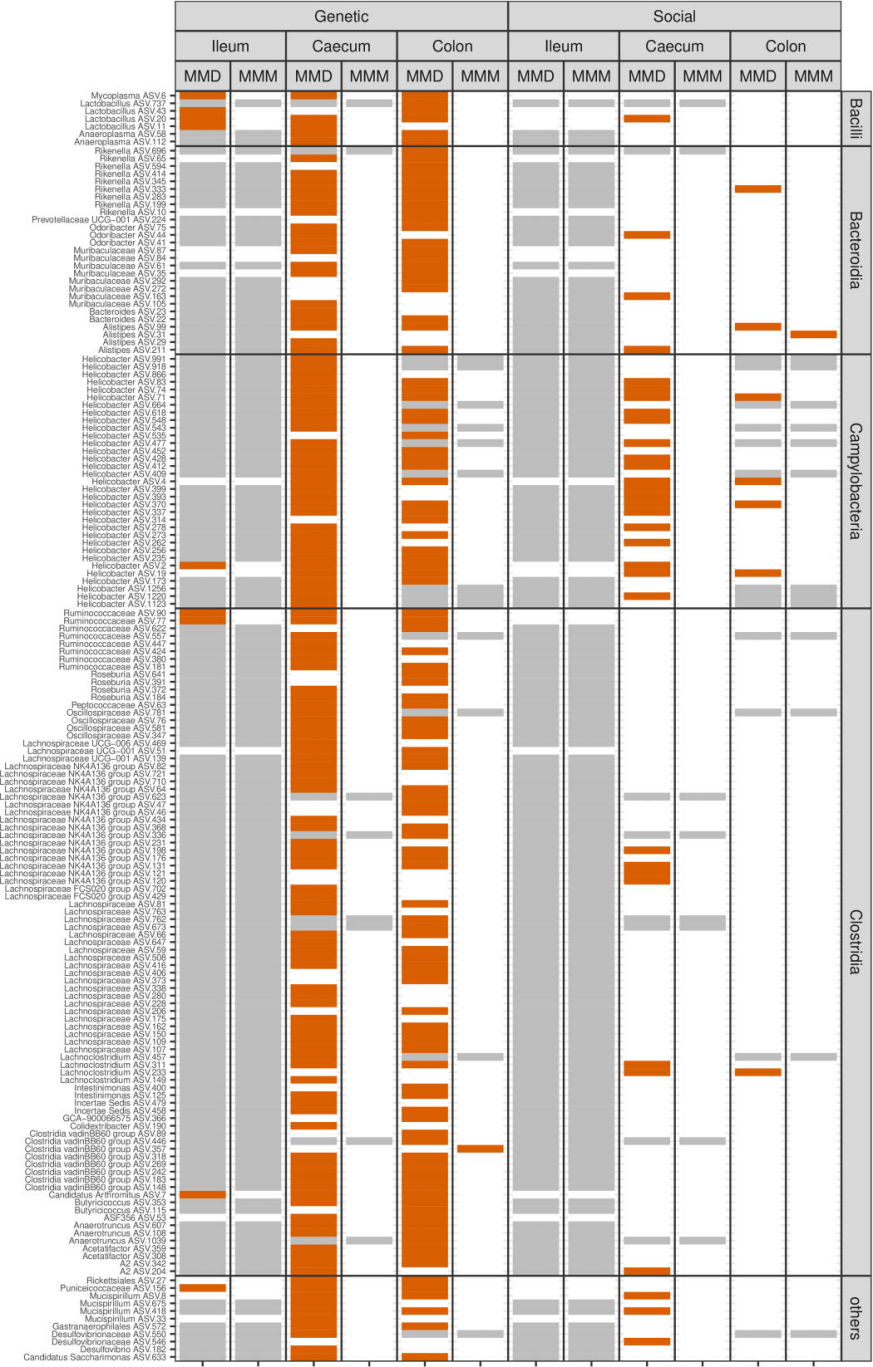
Effect of social interactions and genetic relatedness on abundance variation of bacterial ASVs. The figure shows ASVs whose variation between individuals was significantly (FDR < 0.05) correlated with genetic relatedness or social interactions in red, nonsignificant associations in white, and ASVs not included in the analyses due to their low incidence in specific gut segments (*N* < 20) in gray. Separate analyses were performed for each gut segment (ileum, cecum, and colon) and mouse subspecies (MMM, MMD).

A greater number of ASVs were significantly associated with genetic relatedness and social interactions in MMD than in MMM (Fig. 2). In the case of genetic relatedness, the difference between subspecies can be explained by the overall lower effect of relatedness on the variation in ASVs abundance in MMM (assessed by paired Wilcoxon test for Procrustes sums of squares; *P* < 0.0001 for all gut sections). In the case of the social interaction, Procrustes sums of squares values for each ASV did not differ between MMD and MMM for the colon (*P* = 0.1094) and cecum (*P* = 0.3278), while they were slightly lower for the MMM ileum microbiota (*P* = 0.0221). This suggests that the lower number of significant associations in the MMM cannot be explained by the reduced role of social contacts in this subspecies and is therefore probably due to lower statistical power.

ASVs whose abundance correlated with the genetic relatedness of their hosts were found across a wide range of taxa, including primarily Clostridia, Bacteroidia, Campylobacteria, and to a lesser extent Bacilli. On the other hand, ASVs associated with social transmission were predominantly from the genus *Helicobacter*, with 18 ASVs showing a significant association with social contact intensity (Fig. 2). In addition to *Helicobacter*, social contacts were correlated with the abundance of 17 other ASVs belonging to the genera *Mucispirillum, Lactobacillus, Alistipes, Odoribacter, Lachnoclostridium*, and *Rikenella*, as well as the Lachnospiraceae, the Muribaculaceae, and the Desulfovibrionaceae family.

### Temporal persistence of parental transfer and offspring microbiota similarity

The similarity of microbiota composition between siblings from the same litter exhibited nonlinear changes with age, as evidenced by comparing models that considered linear versus nonlinear trends (Table S4, Supplementary Data). Moreover, there was a significant interaction between mouse age and gut section, indicating that the temporal dynamics of similarity varied along the gut (Table S5, Supplementary Data). Consequently, separate models were fitted for the data from each gut section.

**Figure 3.**
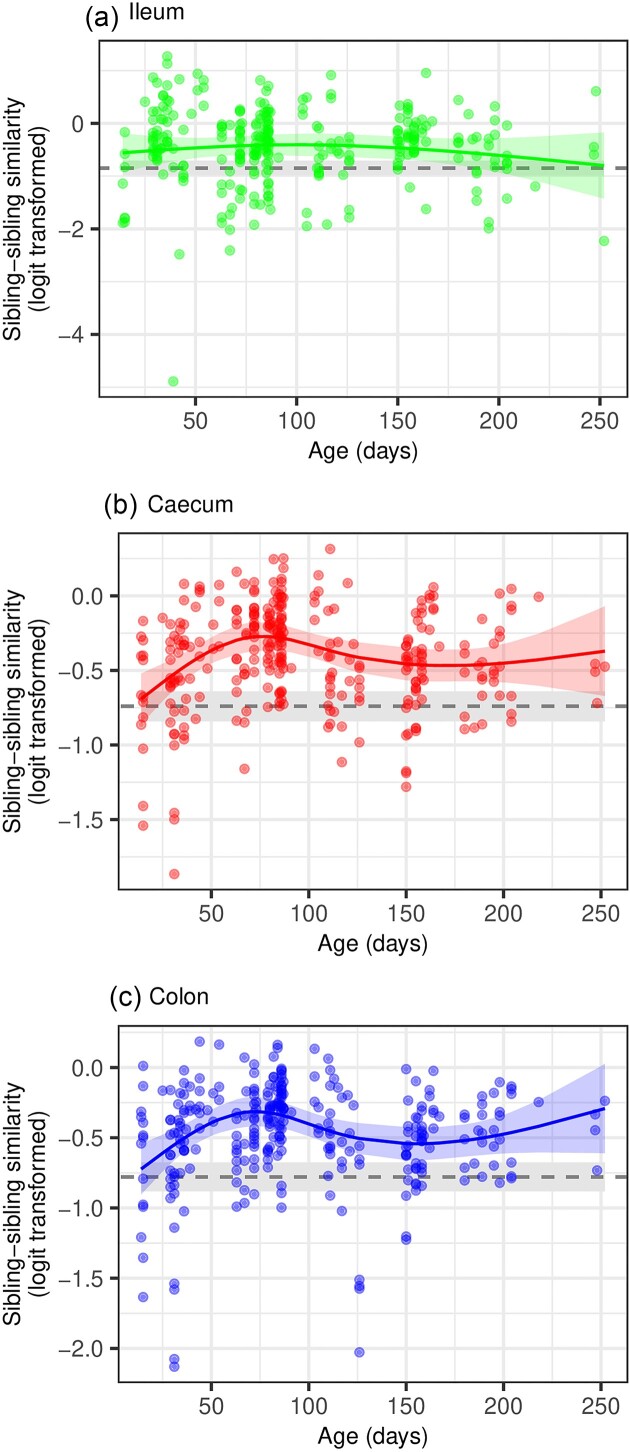
Similarity in microbiota composition between siblings from the same litter. The Bray–Curtis index, which considers the relative abundances of ASVs, was used to examine how the similarity in microbiota between siblings varies on the time scale represented by sibling age. The colored line and shaded area correspond to the mixed model predictions and 95% confidence intervals for each gut section. The gray line and gray shaded area correspond to the average similarity between genetically unrelated individuals (relatedness coefficient ≤ 0.25) and their 95% confidence intervals.

In terms of Bray–Curtis and Jaccard similarity, the microbiota of the colon and cecum showed an initial similarity among siblings at a young age (<20 days old) that was comparable to the population-wide average of unrelated individuals. Subsequently, the microbiota similarity gradually increased until ∼70–80 days of age and then converged back to the population-wide baseline, although it did not reach the baseline even in older mice (>200 days old). The temporal variation in the ileum microbiota was less dramatic, with older siblings showing only slightly increased similarity compared to the population-wide baseline (Fig. 3, Fig. S3, Supplementary Data).

**Figure 4.**
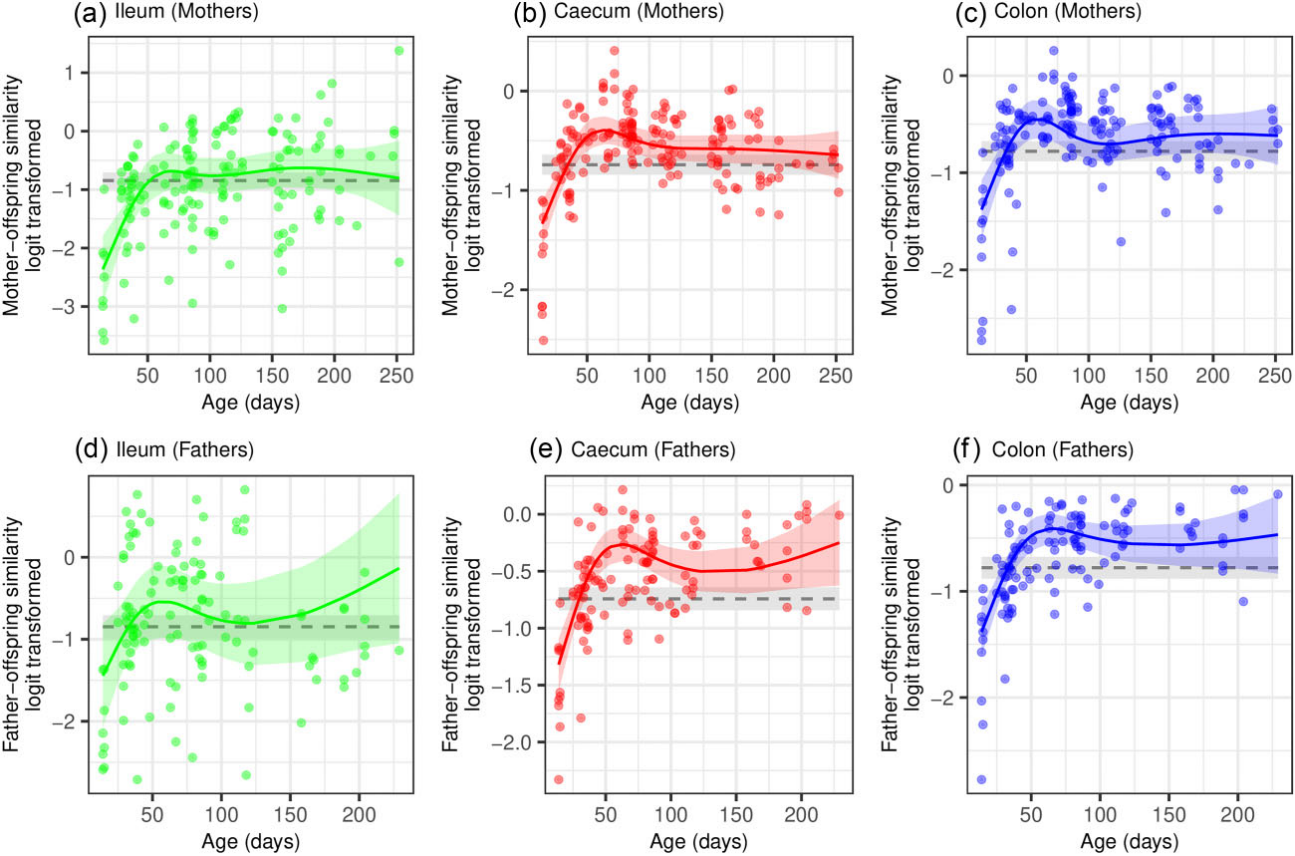
Similarity in microbiota composition between parents and their offspring. The Bray–Curtis index, which considers the relative abundances of ASVs, was used to examine how the similarity in microbiota between parents and offspring varies on the time scale representing offspring age. The colored line and shaded area correspond to the mixed model predictions and 95% confidence intervals for each gut section. The gray line and gray shaded area correspond to the average similarity between genetically unrelated individuals (relatedness coefficient ≤ 0.25) and their 95% confidence intervals.

As with siblings, the microbiota similarity between parents and their genetic offspring also exhibited nonlinear changes with offspring age (Table S4, Supplementary Data), and a significant interaction between offspring age and gut section indicated differential temporal patterns of variation between gut sections (Table S5, Supplementary Data). Age-dependent changes of Bray–Curtis and Jaccard similarity showed consistent patterns, and the results were congruent when analyzing the similarity of offspring to their genetic mothers or fathers (Fig. 4 and Fig. S4, Supplementary Data). The similarity of cecum and colon microbiota between offspring and their parents was highest between 60 and 70 days of age, gradually converging to the population-wide baselines later. In contrast to the lower gut, the confidence intervals for the parent–offspring similarity in ileum microbiota always overlapped with the confidence intervals for the population-wide similarity baseline, regardless of offspring age. The interaction between subspecies and age was significant only in the case of Bray–Curtis similarity between mothers and their offspring, with MMM showing a greater maternal effect than MMD, particularly during the 60–70 day peak (Fig. S5, Supplementary Data). When analyzing the similarities between the microbiota of mother and offspring, significant interaction between the sex and age of the offspring was also found. However, the subsequent model predictions did not reveal a clear pattern (Fig. S5, Supplementary Data).

## Discussion

Our analysis revealed that inter-individual transmission had a significantly smaller effect in the small intestine (ileum) compared to the lower gut (cecum and colon). This difference was observed both at the whole community level and for individual bacterial ASVs. The small intestine exhibited lower alpha diversity and altered taxonomic composition, which has been observed not only in mice (Kreisinger et al. 2015, Suzuki and Nachman 2016, Bendová et al. 2020) but also in other mammalian species (Gresse et al. 2019). These variations are attributed to different environmental conditions determined by the host immune system, high concentrations of bile acids, and differences in metabolic substrates (Zoetendal et al. 2012, Kastl et al. 2020). The microbiota of the small intestine mainly utilizes high-energy substrates of low complexity, whereas the utilization of dietary fiber entering the lower gut favors the development of diversified communities rich in various cross-feeding interactions (Louis et al. 2021).

Indeed, the ileum samples in this study exhibited low taxonomic complexity, with bacteria of only three genera (*Candidatus* Savagella, *Lactobacillus*, and *Mycoplasma*) averaging 80% of the total community and being the dominant component of the ileum microbiota in almost all individuals. The low strength of inter-individual transmission observed for ileum bacteria may be partly due to the low variation in ileum communities. On the other hand, ileal bacteria (including the three genera found in our study) can generally tolerate relatively high oxygen concentrations (Zoetendal et al. 2012, Kastl et al. 2020), and *C*. Savagella forms spores that are excreted in host faeces (Hedblom et al. 2018). While these traits may serve as preadaptations for bacterial transmission through social contact, they can also enable bacterial survival in the external environment. The absence of the transmission signal in the ileum could therefore be a consequence of indirect transmission between individuals, for example, through coprophagy of leftover feces or ingestion of contaminated food or nest material.

Although inter-individual transmission explains only a small portion of the variation in the lower gut microbiota, relatedness and the intensity of social contacts exerted significant independent effects on the microbiota similarity between individuals. The significance of social transmission challenges the seminal study of Moeller et al. (2018), which showed that vertical transmission along host genealogy is by far the most important mechanism shaping gut communities in house mice. This contradiction can be explained by the experimental design of Moeller et al. (2018), which suppressed transmission through social contacts (no inter-individual contacts except for mother and offspring, sibling mating scheme) and through environments (sterile cages), creating a strong *a priori* bias toward vertical transmission. Therefore, it is not surprising that social transmission is significant when studied under more natural conditions.

A substantial difference in transmission patterns was observed between the two house mouse subspecies. Relatedness and social contacts explained a greater portion of variability in the composition of lower gut microbiota and correlated with more bacterial ASVs in MMD compared to MMM. Although this difference could be attributed to variations in the gut microbiota composition between the two subspecies, we find this explanation unlikely. Rather than being the result of a major taxonomic rearrangement involving phylogenetically dissimilar bacterial taxa, the microbiota differences were primarily due to changes in the abundance of closely related bacterial variants (Bendová et al. 2022), which likely depend on similar dispersal strategies.

In this regard, differences in the patterns of social interaction and reproduction between the subspecies (Mikula et al. 2022) appear to be more relevant. Specifically, MMD exhibits a more “demic” social structure, where most social interactions, including mating, occur within cohesive social modules identified with basic reproductive units or demes. This stronger genetic and social structuring of the MMD population also implies a more structured microbiota transmission. Consequently, limited transmission opportunities may predominantly influence microbiota dissimilarity in MMD. In contrast, the ability of bacteria to utilize inter-individual transmission conduits may be more pronounced in the more panmictic population of MMM. In other words, limited inter-individual interactions between demes may restrict microbiota transmission and serve as a major axis of microbiota differentiation in MMD, but not in MMM.

In this experiment, we demonstrated the effects of transmission over the short time scale of several host generations. However, the observed transmission patterns can explain the co-divergence of certain gut bacteria with their host populations/species (Moeller et al. 2016a, Waskito and Yamaoka 2019, Karcher et al. 2020, Suzuki et al. 2022) as a consequence of long-term inter-individual transmission of bacteria through structured mating and social transmission conduits.

At the level of individual bacterial lineages, a notable pattern was the association between multiple ASVs of the genus *Helicobacter* and the intensity of social interactions or relatedness. The dependence of this genus on inter-individual transmission has been observed in human *Helicobacter pylori*, where population genetic variation at the geographic level aligns with ancient human migration routes (Falush et al. 2003, Linz et al. 2007). In wild house mouse populations, the highly efficient transmission of intestinal *Helicobacter* is evident from its extremely high prevalence—virtually all individuals we examined harbored representatives of this genus in their gut microbiota (Wasimuddin et al. 2012, Bendová et al. 2020, Čížková et al. 2024). We have also experimentally demonstrated the easy transmissibility of *Helicobacter* through social contact by co-housing adult laboratory mice lacking *Helicobacter* with individuals from wild populations (Moudra et al. 2021). Given the efficiency of *Helicobacter* transmission, it is likely that colonization of the intestinal mucosa under natural conditions occurs early in the life of mice, which may lead to a priority effect (Debray et al. 2022). This implies that the already occupied mucosal niche can prevent the subsequent incorporation of other *Helicobacter* variants, which is consistent with our previous finding that two closely related *Helicobacter* ASVs exhibit near-perfect specificity for host subspecies under common garden conditions (Bendová et al. 2022). Thus, it appears that efficient inter-individual transmission can maintain microbiota divergence in closely related hosts even in syntopic populations. However, the specificity of mouse subspecies for their “own” *Helicobacter* may also contribute to this pattern.

In addition to *Helicobacter*, numerous other bacterial ASVs from various taxonomic groups have been associated with intensity of social contact or host relatedness, and these associations were consistent between host subspecies. This suggests that some gut bacteria are systematically transmitted between individuals and that these bacteria have different life-history strategies, including both sporulating species (e.g. from the classes Bacilli and Clostridia) and non-sporulating species (e.g. from the class Bacteroidia).

Knowing the age of the individuals in the population studied allowed us not only to quantify the overall effect of parental transmission on the gut microbiota, but also to assess how its magnitude changes from birth through the period of parental care to adulthood. On the timescale corresponding to the age of the offspring, the similarity of the microbiota between parents and offspring showed non-linear trajectories that differed significantly between gut sections. Compared to the lower gut, the similarity of the ileum microbiota between parents and offspring displayed a less pronounced age-dependent pattern. The two lower gut sections showed comparable trajectories. During the breastfeeding [i.e. before the age of ∼23 days; (König and Markl 1987)], the microbiota of the colon and the cecum exhibited a low similarity with that of the parents, which was even lower than the similarity between unrelated individuals. This can be attributed to the influence of breast milk, which shapes the juvenile microbiota through pre- and probiotic compounds, antimicrobial factors or immunoglobulins (van den Elsen et al. 2019), resulting in a distinct composition compared to that of adults (Wernroth et al. 2022). After weaning, the lower gut microbiota of the offspring gradually converged toward that of their parents. The similarity was highest at 60–70 days of age, which corresponds approximately to the time when offspring leave parental territory (van Zegeren 1979, Groó et al. 2013), after reaching full independence from parental care and sexual maturity (at ∼35–40 days of age, [Latham and Mason 2004 et al. 2004, Hiadlovská et al. 2015)]. Thereafter, the similarity of the lower gut microbiota between parents and offspring began to decline. At ∼100 days of age, it became statistically indistinguishable from microbiota similarity between unrelated individuals, although it was still higher on average than the population-wide baseline. These patterns are consistent with the results of a recent meta-analysis of human gut metagenomes (Valles-Colomer et al. 2023), which showed a decrease in the effects of parental transmission with offspring age.

Somewhat surprising was the comparable influence of both parents on the microbiota of their offspring. Considering the maternal transmission during birth and the closer contact with the offspring during parental care (König and Markl 1987), this pattern suggests the role of inherited genetic factors regulating the proliferation of transmitted bacteria in the gut. However, it appears that these effects were less important compared to the impact of inter-individual transmission, as it is unlikely that genetic inheritance explains the peak of microbiota similarity at a specific age of the offspring. We found partial support for subspecies-specific variation in parental effects, as MMM offspring exhibited greater similarity of microbiota with their mother between ∼60–70 days of life compared to MMD.

The transmission of microbiota from parents to offspring should also be reflected in the similarity of microbiota between siblings. Consistent with this expectation, the age-dependent patterns of microbiota similarity between parents and offspring and between siblings were largely congruent. The most striking difference was a persistent increase in sibling similarity relative to the population baseline. This pattern suggests the presence of family-specific effects that have a long-term impact on the composition of the microbiota of the entire litter. For example, factors such as maternally or paternally induced stress in the offspring (Kemp et al. 2021) or other epigenetic factors such as a higher frequency of coprophagy in young mice compared to adults (Ebino et al. 1987), could contribute to these effects. However, it is also possible that the high similarity of the siblings compared to the baseline is partly due to the fact that the sampled siblings were the same age, which was not the case in the baseline population or the parent–offspring pairs.

In conclusion, our findings demonstrate that inter-individual transmission, mediated either by the transmission of microbiota along genealogical lineages or by social contacts between conspecifics, has significant effects on the composition of the gut microbiota of the house mouse bred under semi-natural conditions. However, these effects are not consistent throughout the gastrointestinal tract, with inter-individual transmission being notably weaker in the small intestine than in the lower gut. Importantly, the relative strength of microbiota transmission from parents varies greatly depending on the age of the offspring. Failure to account for these temporal dynamics can introduce strong biases in the quantification of parental microbiota transmission and affect consistency in replicated studies conducted with the same species.

Furthermore, we identified bacterial taxa that exhibited consistent transmission patterns between the two mouse subspecies, indicating their general ability for inter-individual transmission. Despite the similarity in the transmitted bacteria, we observed clear differences in the strength of microbiota transmission between the two subspecies, likely influenced by variations in their social and reproductive networks. This suggests that the transmission of bacteria among individuals is not only determined by their dispersal abilities, but also by the transmission opportunities mediated by interactions between individuals. These findings have important implications for experiments conducted under laboratory conditions where mating and social contacts between mice are restricted. Although we were able to demonstrate the importance of social interactions in shaping the gut microbiota, our study was not designed to distinguish the relative importance of different types of social encounters. These questions should therefore be clarified by further research.

## Supplementary Material

fiae075_Supplemental_Files

## Data Availability

Sequencing data associated with this project are archived in the European Nucleotide Archive (project accession number: PRJEB72825). Accession numbers for each sample are provided in Table S1, Supplementary Data. Scripts for data analyses are available at https://github.com/jakubkreisinger/HM_soc_gen.
