# Supplementary figures and images for: The strength of gut microbiota transfer along social networks and genealogical lineages in the house mouse
Source: FEMS Microbiol Ecol. 2024 May 10;100(6):fiae075. doi: 10.1093/femsec/fiae075 (PMC11134300; doi:10.1093/femsec/fiae075)

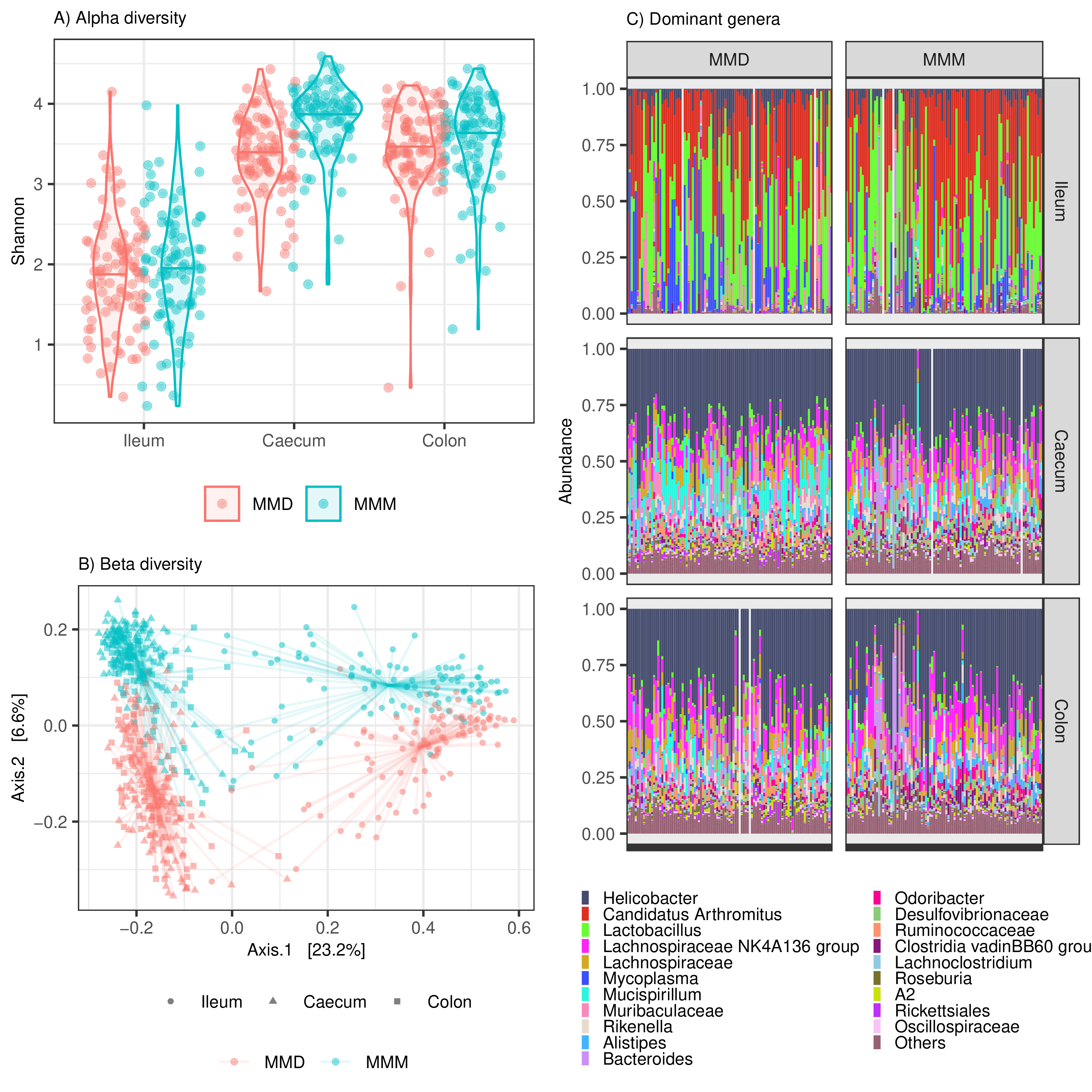

Supplement: fiae075_Supplemental_Files [file fiae075_supplemental_files.zip › supp data Figure.S1.MMM_MMD_summary.jpg]

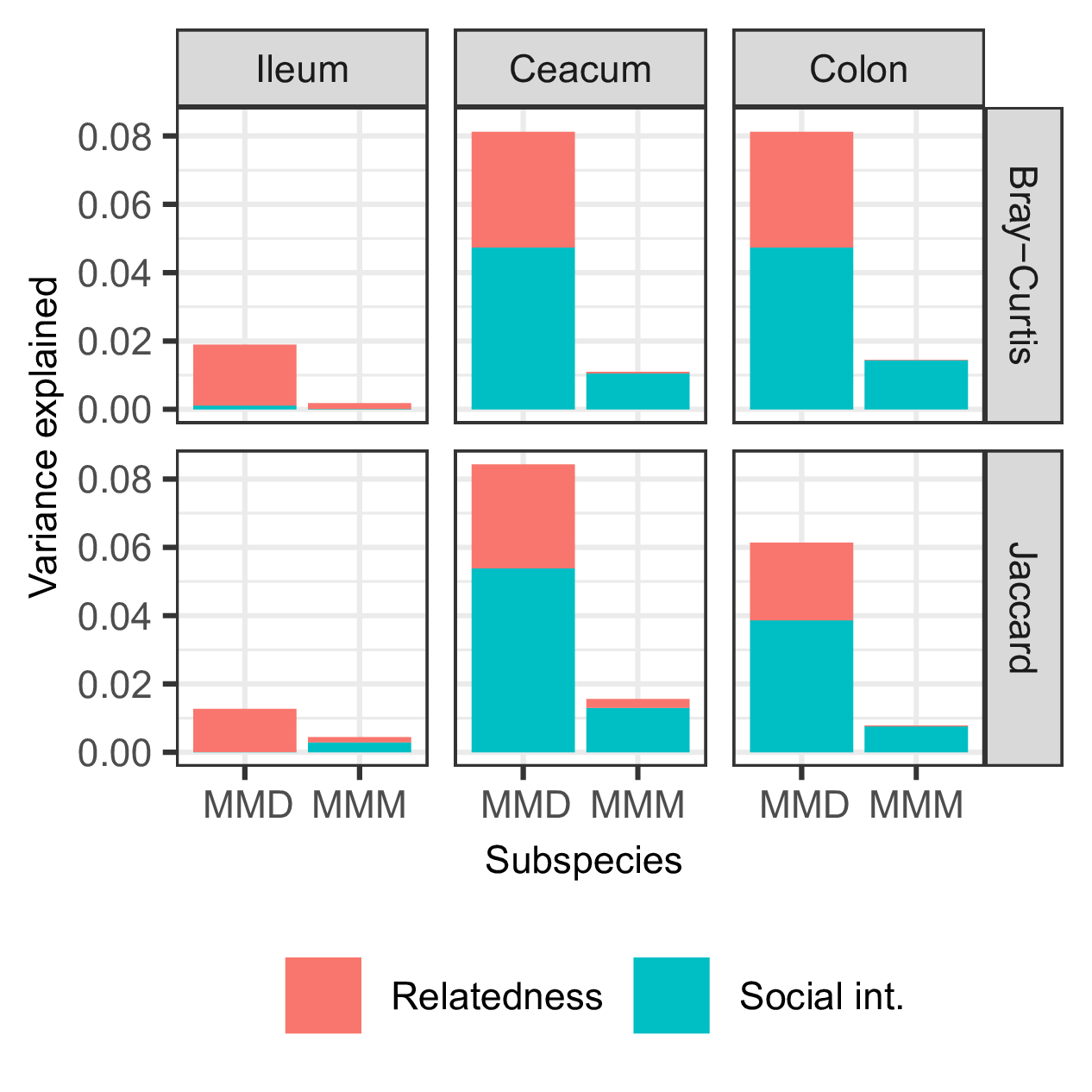

Supplement: fiae075_Supplemental_Files [file fiae075_supplemental_files.zip › supp data Figure.S2.LMER_Var_raw_corrected_unrel_dascorr.jpg]

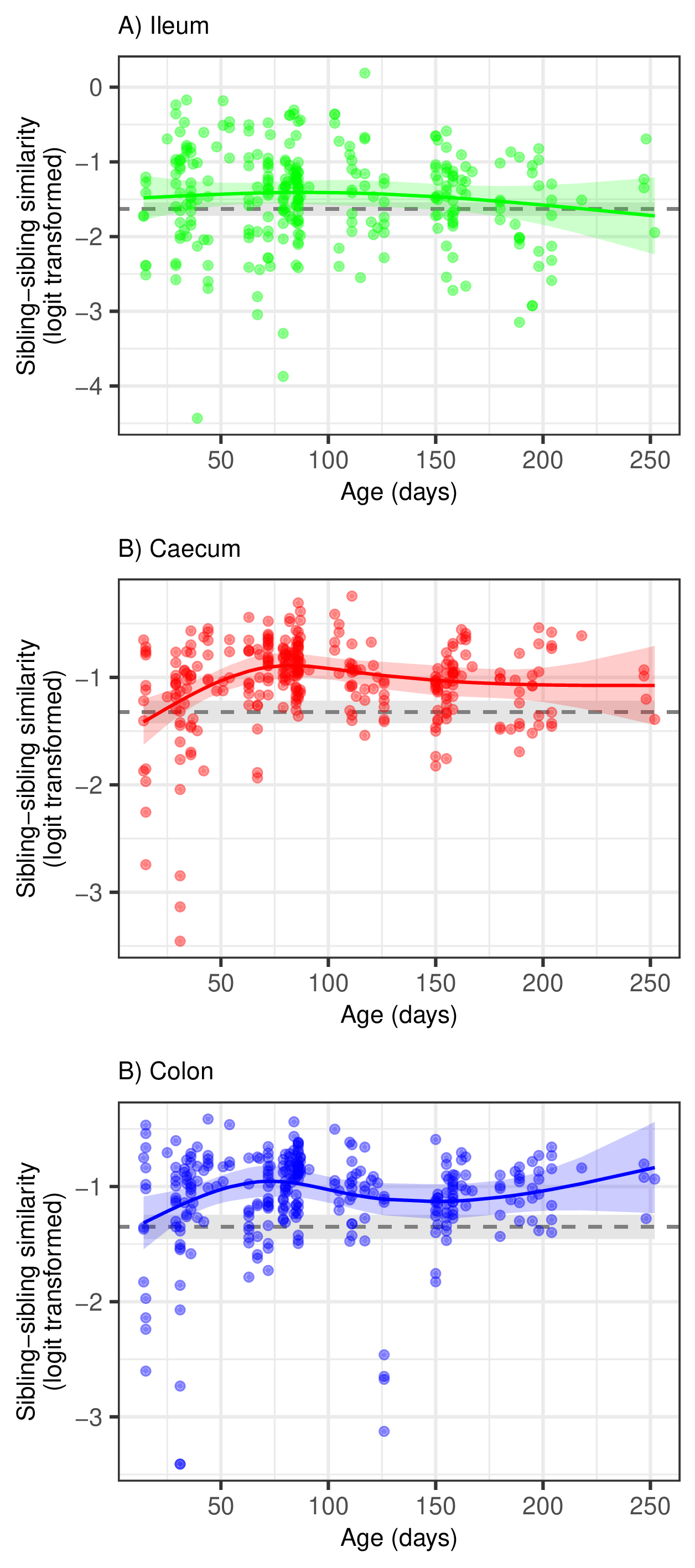

Supplement: fiae075_Supplemental_Files [file fiae075_supplemental_files.zip › supp data Figure.S3.Offspring_offspring_disimilarity.JA_v2b.jpg]

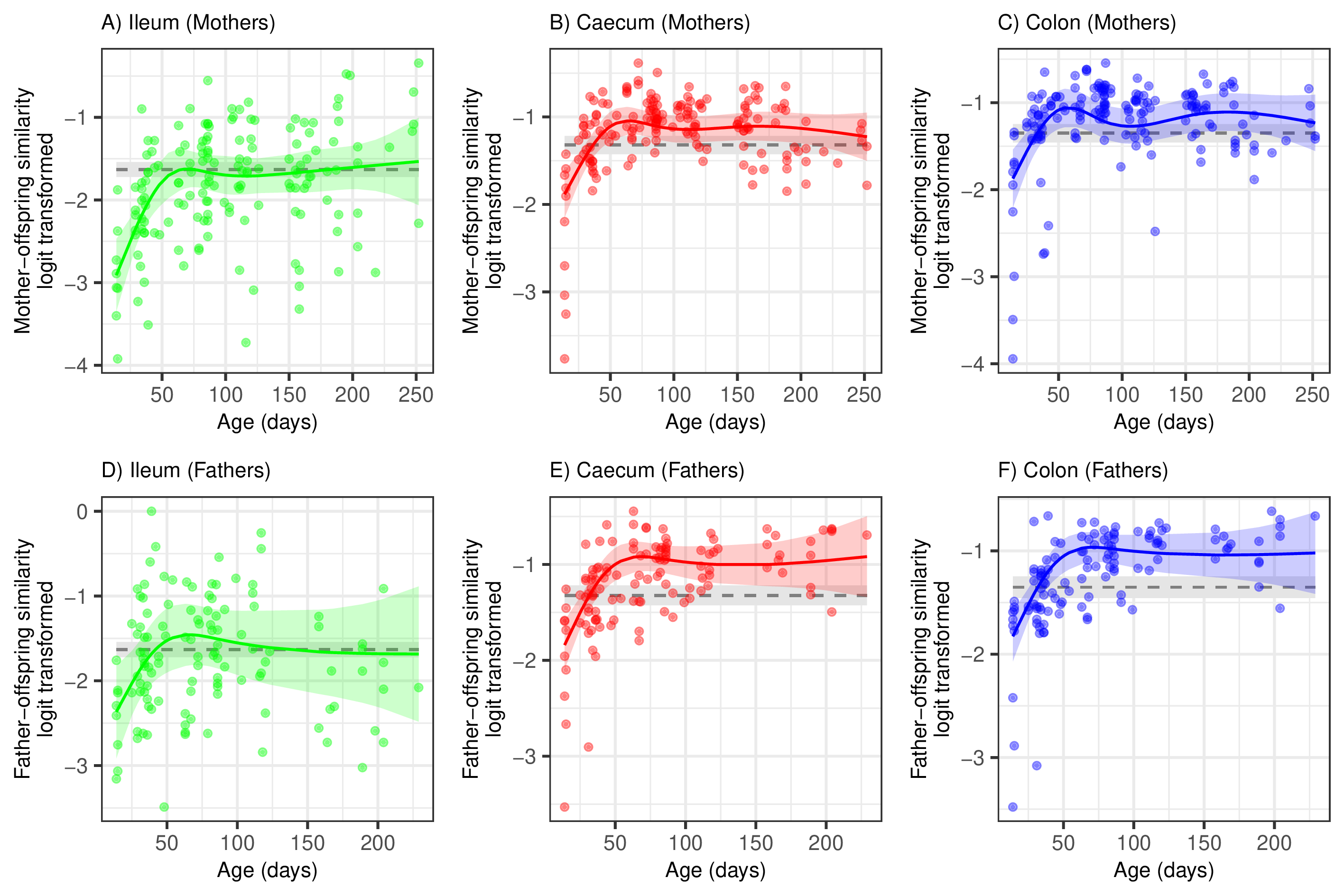

Supplement: fiae075_Supplemental_Files [file fiae075_supplemental_files.zip › supp data Figure.S4.Parent_offspring_disimilarity.JA_v2.jpg]

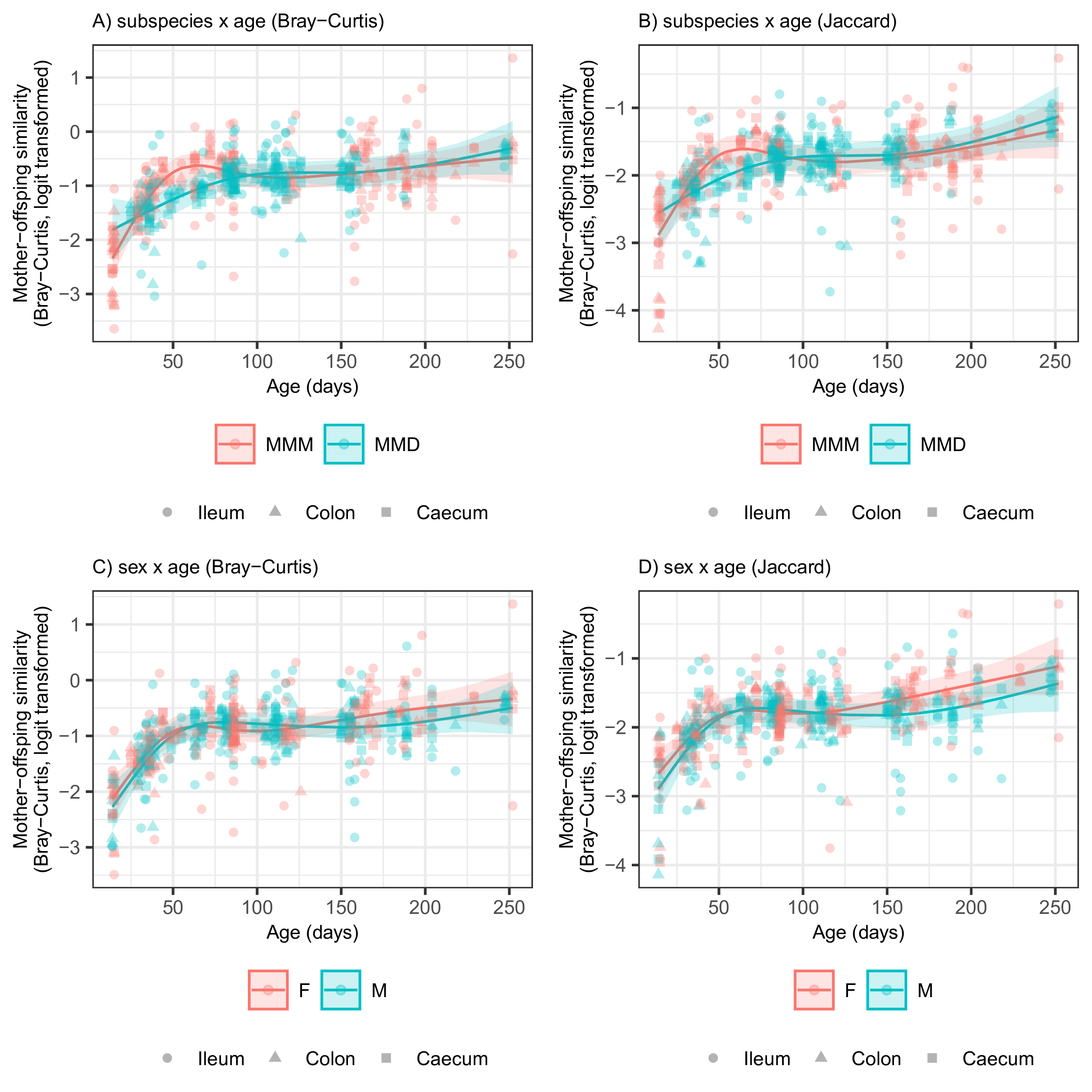

Supplement: fiae075_Supplemental_Files [file fiae075_supplemental_files.zip › supp data Figure.S5.Mother_subspecies_sex_int_dascorr.jpg]
